# Dual-Layer Spectral CT of Pancreas Ductal Adenocarcinoma: Can Virtual Monoenergetic Images of the Portal Venous Phase Be an Alternative to the Pancreatic-Phase Scan?

**DOI:** 10.5334/jbsr.2798

**Published:** 2022-09-22

**Authors:** Yeo Eun Han, Beom Jin Park, Deuk Jae Sung, Min Ju Kim, Na Yeon Han, Ki Choon Sim, Yongwon Cho, Hayeon Kim

**Affiliations:** 1 Korea University Anam Hospital, 73 Goryeodae-ro, Seongbuk-gu, Seoul, 02841, KR; 2 Korea University Guro Hospital, 148 Gurodong-ro, Guro-gu, Seoul, 08308, KR

**Keywords:** Pancreatic carcinoma, pancreatic neoplasms, computed tomography, radiation, spectral computed tomography

## Abstract

**Objectives:**

To determine the performance of virtual monoenergetic images (VMIs) of the portal venous phase (PVP) compared with the pancreatic-phase image for pancreatic ductal adenocarcinoma (PDAC) evaluation.

**Materials and methods:**

This retrospective study enrolled 64 patients with PDAC who underwent pancreatic CT with dual-layer spectral CT between February 2018 and January 2020. A polychromatic pancreatic-phase image and VMIs at 40 (VMI_40_), 55 (VMI_55_), and 70 keV (VMI_70_) of the PVP were generated. The tumor-to-pancreas contrast-to-noise ratio (CNR), attenuation difference, peripancreatic vascular signal-to-noise ratio (SNR), and CNR were compared among the four images. Subjective image analysis was performed for tumor conspicuity, heterogeneity, size, and arterial invasion.

**Results:**

VMI_40_ and VMI_55_ demonstrated higher tumor-to-pancreas CNR, attenuation difference, and higher peripancreatic vascular CNR and SNR than the pancreatic-phase image and VMI_70_ (p < .001). On subjective analysis, VMI_55_ showed the best tumor conspicuity. Moreover, the inter-reader agreement for arterial invasion in VMIs from the PVP was not inferior to that in the pancreatic-phase image.

**Conclusion:**

For evaluating PDAC, the VMI_55_ of the PVP was superior to the pancreatic-phase image in terms of tumor conspicuity and peripancreatic vascular enhancement. Therefore, the VMI_55_ of the PVP could be an alternative to the pancreatic-phase scan in patients suspicious of PDAC.

## Introduction

Pancreatic ductal adenocarcinoma (PDAC) is the most devastating pancreatic disease, and detection of PDAC is a critical goal of the pancreatic CT. However, the symptom of PDAC is non-specific, and a large number of diseases need to be differentiated [1]. The pancreatic-phase is not necessary for all differential diagnoses. Considering the low incidence of pancreatic cancer, the routine acquisition of pancreatic-phase in all patients having symptom which is suspicious for PDAC will result in unnecessary radiation exposure.

The pancreatic-phase provides better tumor conspicuity and peripancreatic arterial enhancement than the portal venous phase (PVP) [2,3]. If additional images reconstructed from the PVP achieve equivalent tumor conspicuity and peripancreatic arterial enhancement with pancreatic-phase, a single-phase study will be enough for the patient with non-specific symptoms suspicious for PDAC. The introduction of spectral CT has enabled the acquisition of virtual monoenergetic images (VMIs) using post-processing algorithms. The K-edge of iodine is 33 keV. As the monoenergetic energy level approaches 33 keV, the image contrast achieved by the administration of an intravenous contrast medium is maximized because of the enhanced photoelectric effect [4]. Previous studies have revealed that low-keV VMI improves the PDAC detection [5,6,7,8]. Among the multiple ways to acquire spectral data, dual-layer detector-based spectral CT is the newest commercially available technique and has the advantage that spectral data are obtained for all scans retroactively [9]. Therefore, this study aimed to determine whether VMIs based on dual-layer spectral CT from the PVP could replace the pancreatic-phase scan to detect PDAC. For this purpose, we evaluated tumor conspicuity and peripancreatic arterial enhancement in VMIs of the PVP compared with those in the pancreatic-phase image in patients with PDAC, using both objective and subjective parameters.

## Materials and Methods

### Study Participants

Our institutional review board approved this retrospective study (registry no. 2018AN0448) and waived the requirement for its informed consent.

**Figure 1:**
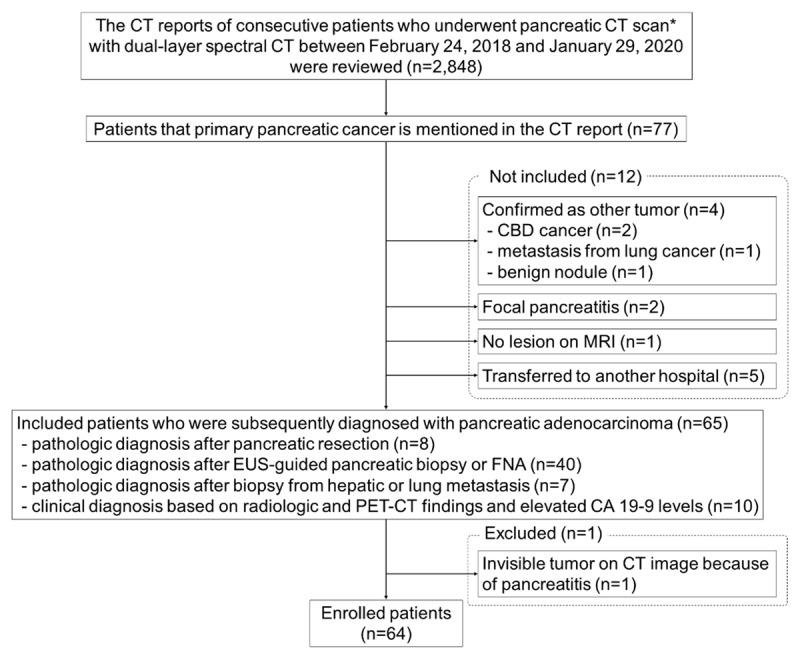
Flow diagram of study participants. * Comprising nonenhanced, pancreatic, and portal venous phases. CBD: common bile duct; EUS: endoscopic ultrasound; FNA: fine-needle aspiration; CA: cancer antigen.

The flow diagram of study participants was demonstrated in Figure 1. The inclusion criteria were PDAC patients who underwent pancreatic CT scan with dual-layer spectral CT. For patient inclusion, the CT reports of 2,848 consecutive patients who underwent pancreatic CT using dual-layer spectral CT between February 24, 2018 and January 29, 2020 were reviewed. Including 65 patients, who were subsequently diagnosed with PDAC. The exclusion criteria were poor tumor conspicuity on CT scan for image analysis, and one patient was excluded because the tumor was not visualized on CT image owing to pancreatitis.

### CT Protocol and Image Reconstruction

All CT scans were acquired using a dual-layer spectral CT (IQon Spectral CT; Philips Healthcare, Cleveland, OH). Pancreatic CT comprised a non-enhanced phase, a pancreatic-phase, and a PVP. Intravenous contrast medium (Iomeron 350; Bracco, Milan, Italy) was injected at a rate of 3 mL/s for a total amount of 2 mL/kg body weight using a power injector system. The bolus tracking method was applied, and the pancreatic-phase scan started 27s after the abdominal aorta reached 150 HU. The PVP scan started 50s after the abdominal aorta reached 150 HU. The upper abdomen was scanned in the non-enhanced and pancreatic-phases, and the whole abdomen and pelvis were scanned in the PVP. After the CT evaluation, the conventional polychromatic images of the pancreatic-phase at 120 kVp, VMIs of the PVP at 40 keV (VMI_40_), 55 keV (VMI_55_), and 70 keV (VMI_70_) were reconstructed. More detailed information is provided in supplementary file 1: Appendix. Materials and methods.

### Image Analysis

For objective analysis, one radiologist with five years of experience analyzed the images. Circular regions of interest (ROIs) were drawn in the normal pancreatic parenchyma, tumor, paravertebral muscle, celiac trunk, portal vein, superior mesenteric artery (SMA), and superior mesenteric vein (SMV) in the four reconstructed images. The average attenuation (AV) and standard deviation (SD) of the Hounsfield unit (HU) were recorded for each ROI.

The conspicuity of PDAC was evaluated using the tumor-to-pancreas CNR and attenuation difference.


\begin{array}{l}
{\rm{CNR}} = \frac{{A{V_P} - A{V_T}}}{{\left( {S{D_P} + S{D_T}} \right)/2}}\,\,\\
{\rm{Attenuation\;difference}} = A{V_P} - A{V_T}
\end{array}

AV_P_ and SD_P_ are variables of the normal pancreatic parenchyma, and AV_T_ and SD_T_ are tumor variables.

Peripancreatic vasculature enhancement was evaluated using SNR and CNR.


\begin{array}{l}
{\rm{SNR}} = \frac{{A{V_{VS}}}}{{S{D_{VS}}}}\\
{\rm{CNR}} = \frac{{A{V_{VS}} - A{V_{PM}}}}{{S{D_{PM}}}}
\end{array}

AV_VS_ and SD_VS_ are vessel variables, and AV_PM_ and SD_PM_ are variables of the paravertebral muscle.

For subjective analysis, two radiologists (5 and 20 years of experience) independently analyzed the images and evaluated subjective tumor conspicuity, heterogeneity, size, and arterial invasion. More detailed methods for images analysis are provided in supplementary file 1: Appendix. Materials and methods.

### Statistical Analyses

The Wilcoxon signed-rank test and paired t-test were used to compare objective parameters and subjective tumor conspicuity. Cohen’s simple kappa test was used to evaluate the inter-reader agreement of subjective parameters. Bonferroni correction was performed for multiple comparisons. All statistical analyses were performed using the SPSS software (version 25; SPSS). More detailed information is provided in supplementary file 1: Appendix. Materials and methods.

## Results

### Study Participants

A total of 64 patients (30 men, 34 women) were enrolled in this study and seven of whom underwent surgery. The mean age was 69 ± 10 years in all patients.

### Objective Conspicuity of PDAC

**Figure 2:**
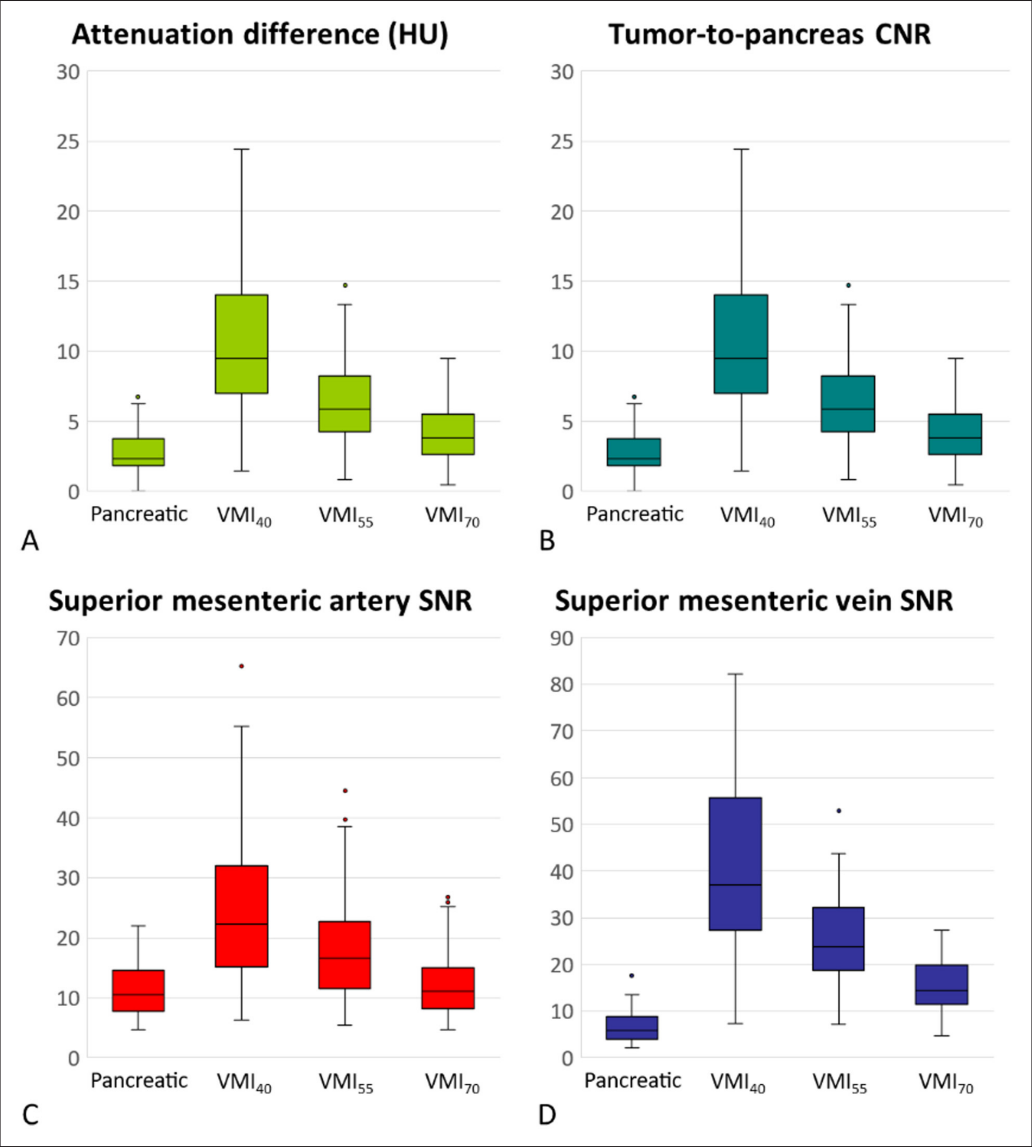
Box whisker plot of objective parameters for evaluating pancreatic ductal adenocarcinoma on multi-reconstructed spectral CT images. HU: Hounsfield unit; CNR: contrast-to-noise ratio; SNR: signal-to-noise ratio; Pancreatic: pancreatic-phase; VMI_40, 55, 70_: virtual monoenergetic image at 40, 55, and 70 keV of the portal venous phase.

**Figure 3:**
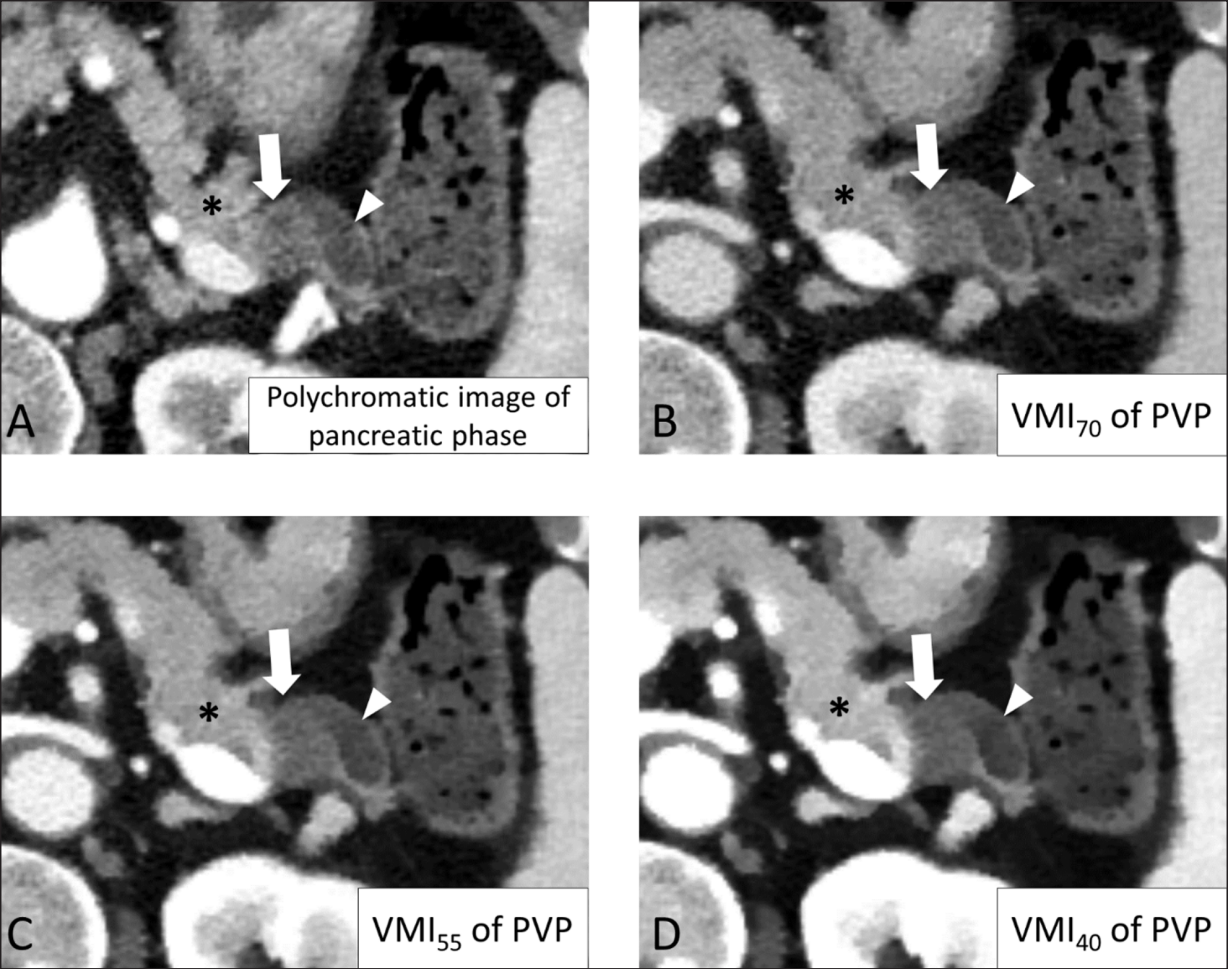
CT images of a 72-year-old woman with pancreatic ductal adenocarcinoma (PDAC). Among the four reconstructed images, VMI_55_ **(C)** and VMI_40_ **(D)** showed better contrast between the PDAC (arrow), pancreatic parenchyma (asterisk), and pancreatic duct (arrowhead) than the conventional pancreatic phase **(A)** and VMI_70_ **(B)**. PVP: portal venous phase; VMI_40, 55, 70_: virtual monoenergetic image at 40, 55, and 70 keV of the PVP.

**Figure 4:**
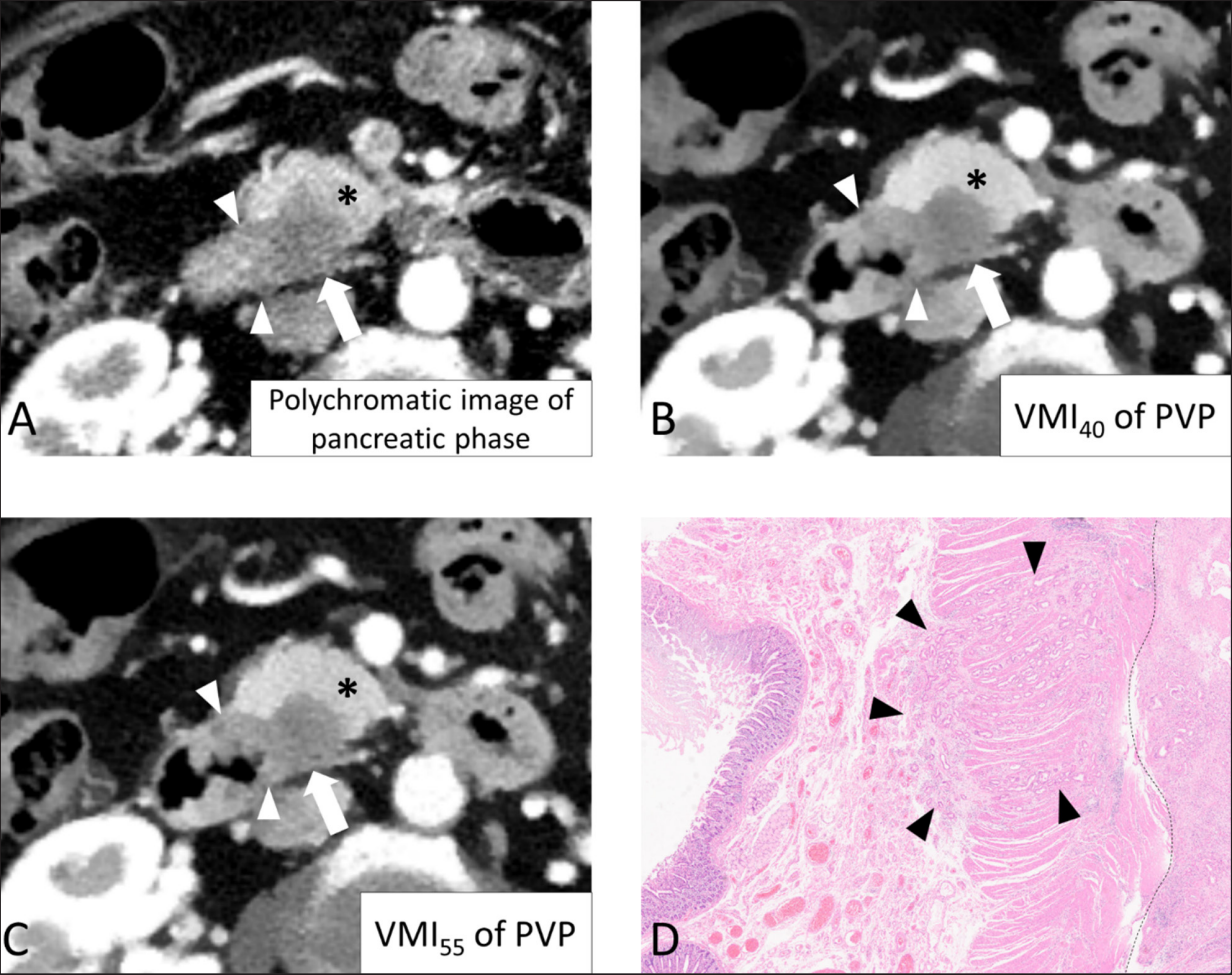
CT images of a 70-year-old woman with pancreatic ductal adenocarcinoma (PDAC). Among the three reconstructed images, VMI_40_ **(B)** and VMI_55_ of PVP **(C)** showed better contrast between the PDAC (arrow) and pancreatic parenchyma (asterisk). The invasion of the duodenum by PDAC is more clearly visualized on VMI_40_ (B) and VMI_55_ (C) of the PVP than on the conventional pancreatic phase **(A). (D)** On pathologic evaluation, PDAC invaded (arrowhead) the duodenum (left side of the dashed line) through the muscularis propria and submucosa (hematoxylin and eosin stain, ×2). PVP: portal venous phase; VMI_40, 55, 70_: virtual monoenergetic image at 40, 55, and 70 keV of the PVP.

The tumor-to-pancreas attenuation difference and tumor-to-pancreas CNR in each reconstructed image are detailed in Table 1 and Figure 2. The p-values of pairwise comparisons of all objective parameters among the four reconstructed images are shown in supplementary file 2: Appendix. P values. VMI_40_ demonstrated a higher tumor-to-pancreas attenuation difference and CNR than the pancreatic-phase image, VMI_55_, and VMI_70_. VMI_55_ demonstrated a higher tumor-to-pancreas attenuation difference and CNR than the pancreatic-phase image and VMI_70_. The tumor-to-pancreas attenuation difference and CNR indicated that VMI_40_ and VMI_55_ showed better objective tumor conspicuity than the pancreatic-phase image (Figures 3 and 4).

**Table 1:** Objective parameters for evaluating pancreatic ductal adenocarcinoma on multi-reconstructed spectral CT images.


PARAMETERS	PANCREATIC	VMI_40_	VMI_55_	VMI_70_

Tumor-to-pancreas attenuation difference (HU)	77.6 ± 35.9	250.2 ± 115.5	131.5 ± 59.4	78.8 ± 37.4

Tumor-to-pancreas CNR	2.7 ± 1.4	10.6 ± 5.5	6.6 ± 3.3	4.2 ± 2.2

SNR				

Celiac trunk	10.1 ± 3.7	26.6 ± 10.9	17.7 ± 6.5	11.9 ± 3.6

Portal vein	7.2 ± 3.5	41.4 ± 15.0	24.3 ± 6.8	15.3 ± 3.8

SMA	11.2 ± 4.4	24.5 ± 12.4	17.7 ± 8.6	12.1 ± 5.1

SMV	6.4 ± 3.1	41.2 ± 20.1	24.8 ± 9.5	15.5 ± 5.4

CNR				

Celiac trunk	16.2 ± 6.0	50.6 ± 19.9	26.8 ± 12.8	15.9 ± 6.7

Portal vein	8.1 ± 4.1	62.1 ± 20.6	32.0 ± 12.5	19.0 ± 7.6

SMA	17.9 ± 7.7	52.3 ± 41.2	28.8 ± 13.3	16.8 ± 7.2

SMV	7.3 ± 5.1	60.1 ± 29.4	33.1 ± 15.3	19.2 ± 8.1


Pancreatic: pancreatic-phase; VMI_40, 55, 70_: virtual monoenergetic image at 40, 55, and 70 keV of the portal venous phase; HU: Hounsfield unit; CNR: contrast-to-noise ratio; SNR: signal-to-noise ratio; SMA: superior mesenteric artery; SMV: superior mesenteric vein.

### Peripancreatic Vascular Enhancement

**Figure 5:**
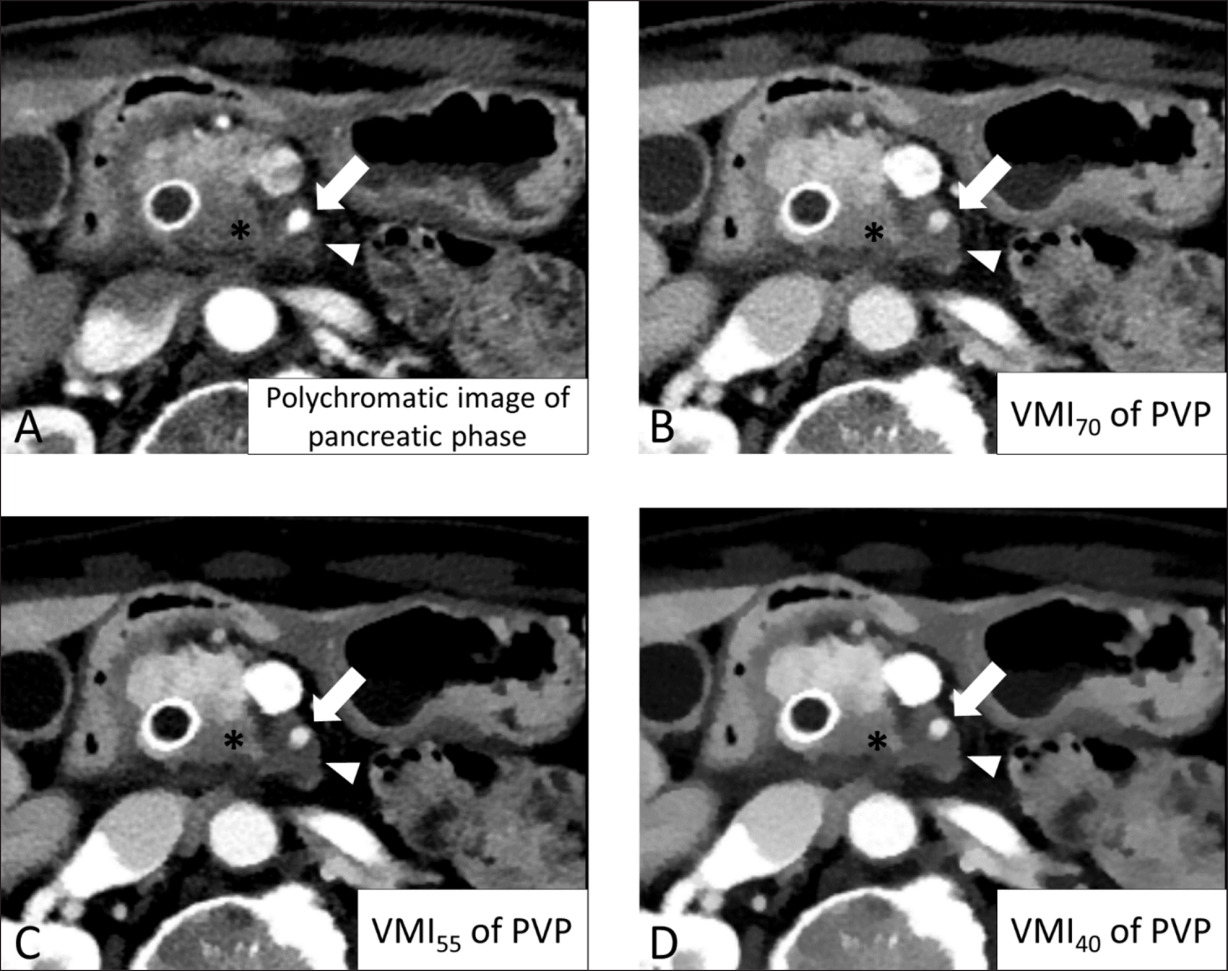
CT images of a 68-year-old man with pancreatic ductal adenocarcinoma (PDAC). The superior mesenteric artery (arrow) is well visualized on both VMI_40_ **(B)** and VMI_55_ of PVP **(C)**, as well as on the pancreatic phase **(A)**. PDAC (asterisk) and adjacent fat are more clearly demarcated on VMI_40_ (B) and VMI_55_ **(C)** of the PVP than on the pancreatic phase (A). The extent of tumor contact with the superior mesenteric artery (arrowhead) is more clearly visualized on VMI_40_ (B) and VMI_55_ (C) of the PVP than on the pancreatic phase. PVP: portal venous phase; VMI_40, 55, 70_: virtual monoenergetic image at 40, 55, and 70 keV of the PVP.

The peripancreatic vascular SNR and CNR of each reconstructed image are detailed in Table 1 and Figure 2. In the assessment of the celiac trunk and SMA, VMI_40_ demonstrated a higher SNR and CNR than the pancreatic-phase image, VMI_55_, and VMI_70_. VMI_55_ demonstrated higher SNR and CNR than the pancreatic-phase image and VMI_70_. The SNR and CNR of the celiac trunk and SMA indicated that VMI_40_ and VMI_55_ showed better peripancreatic arterial enhancement than the pancreatic-phase image (Figure 5).

In the assessment of the portal vein and SMV, VMI_40_ demonstrated higher SNR and CNR than the pancreatic-phase image, VMI_55_, and VMI_70_. VMI_55_ demonstrated a higher SNR and CNR than the pancreatic-phase image and VMI_70_. The SNR and CNR of the portal vein and SMV indicated that VMI_40_ and VMI_55_ showed better peripancreatic venous enhancement than the pancreatic-phase image.

### Subjective Image Analysis

The results of the analysis of subjective parameters and inter-reader agreement (Cohen’s simple kappa) are detailed in Table 2, and the p-values of pairwise comparisons of parameters between the four reconstructed images are detailed in supplementary file 2: Appendix. P values. Subjective tumor conspicuity was the highest (p ≤ .007) in VMI_55_ for both readers (3.31 ± 0.69 and 2.92 ± 0.95).

**Table 2:** Subjective parameters for evaluating pancreatic ductal adenocarcinoma on multi-reconstructed spectral CT images.


SUBJECTIVE PARAMETERS	READER 1	READER 2	KAPPA

Tumor conspicuity			

Pancreatic	2.97 ± 0.93	2.48 ± 0.80	0.24

VMI_40_	2.44 ± 0.77	2.69 ± 0.96	0.16

VMI_55_	3.31 ± 0.69	2.92 ± 0.95	0.16

VMI_70_	2.61 ± 0.70	2.58 ± 0.92	0.36

Tumor heterogeneity			

Pancreatic	0.66 ± 0.72	0.77 ± 0.68	0.49

VMI_40_	1.05 ± 0.68	1.31 ± 0.59	0.28

VMI_55_	1.00 ± 0.71	1.27 ± 0.62	0.41

VMI_70_	0.88 ± 0.75	1.11 ± 0.59	0.48

Tumor size (cm)			

Pancreatic	4.19 ± 1.73	4.45 ± 1.75	N/A

VMI_40_	4.62 ± 2.09	4.79 ± 1.81	N/A

VMI_55_	4.56 ± 2.11	4.51 ± 1.76	N/A

VMI_70_	4.44 ± 2.17	4.62 ± 1.77	N/A

Celiac trunk invasion			

Pancreatic	0.63 ± 0.87	0.48 ± 0.78	0.72

VMI_40_	0.67 ± 0.91	0.67 ± 0.87	0.77

VMI_55_	0.63 ± 0.90	0.63 ± 0.86	0.76

VMI_70_	0.59 ± 0.90	0.58 ± 0.85	0.77

Superior mesenteric artery invasion			

Pancreatic	0.67 ± 0.86	0.66 ± 0.78	0.71

VMI_40_	0.80 ± 0.89	0.78 ± 0.85	0.82

VMI_55_	0.83 ± 0.88	0.80 ± 0.86	0.75

VMI_70_	0.73 ± 0.88	0.73 ± 0.84	0.79

Splenic artery invasion			

Pancreatic	0.80 ± 0.89	0.91 ± 0.90	0.76

VMI_40_	1.13 ± 0.93	1.13 ± 0.90	0.80

VMI_55_	1.13 ± 0.93	1.09 ± 0.90	0.78

VMI_70_	1.05 ± 0.92	1.02 ± 0.90	0.79


Pancreatic: pancreatic-phase; VMI_40, 55, 70_: virtual monoenergetic image at 40, 55, and 70 keV of the portal venous phase; N/A: not available.

Tumor size, heterogeneity, and arterial invasion on VMIs of the PVP were compared with those on the pancreatic-phase image. The tumor sizes were slightly larger on VMI_40_ than on the pancreatic-phase image for both readers and on VMI_55_ than on the pancreatic-phase image for reader 1. The grades of SMA and splenic arterial invasion were higher on VMI_40_ and VMI_55_ than on the pancreatic-phase image for both readers. The grade of celiac trunk invasion was higher on VMI_40_ and VMI_55_ than on the pancreatic-phase image for reader 2. The inter-reader agreement for arterial invasion was strong in VMI_40_ for SMA and splenic artery (kappa = 0.82 and 0.80) and moderate in VMI_40_ for celiac trunk (kappa = 0.77). In VMI_55_, VMI_70_, and pancreatic-phase, the inter-reader agreement for arterial invasion was moderate for the celiac trunk, SMA, and splenic artery (kappa = 0.71–0.79).

## Discussion

In this study, VMI_40_ and VMI_55_ of the PVP showed higher objective tumor conspicuity and peritumoral arterial enhancement than pancreatic-phase images. During subjective image analysis, VMI_55_ showed the best tumor conspicuity. Our objective image analysis results were similar to those of a previous study, although the subjective image analysis results differed. Nagayama et al. reported that VMI_40_ and VMI_50_ of the PVP showed better objective tumor conspicuity than pancreatic-phase images. During their subjective image analysis, VMI_40_ of the PVP showed the best tumor conspicuity [6]. However, in our study, VMI_55_ was better than VMI_40_ for subjective tumor conspicuity. Decreasing the VMI reconstruction energy below 70 keV results in not only higher objective tumor conspicuity but also higher VMI noise [4,6]. The combination of higher objective tumor conspicuity and higher image noise might have led to the better subjective tumor conspicuity of VMI_55_ than VMI_40_. This result is consistent with other studies that suggested 50–55 keV as the optimal VMI setting for tumor evaluation [10,11]. Consequently, we recommend 55 keV as the optimal level of VMI acquisition for PDAC evaluation in the PVP.

When evaluating peripancreatic arteries, VMI_40_ and VMI_55_ of the PVP demonstrated higher SNR and CNR than pancreatic-phase images. This is supported by a prior study comparing the SNR and CNR of arteries between arterial-phase images and VMIs of the venous phase. In this study, VMIs at 40–50 keV of the venous phase were not inferior to the arterial-phase image [12]. Nagayama et al. also reported an equivalent arterial depiction between VMI_40_ or VMI_55_ of the PVP and pancreatic-phase images [6].

During subjective image analysis, tumor size and tumor contact with the artery tended to be larger in lower-keV VMIs. A possible explanation for this is that low-keV VMIs more clearly show the difference in attenuation. Therefore, for areas with a mixture of normal structures and tumors, low-keV VMIs show a larger attenuation difference with adjacent normal structures than conventional images. As a result, low-keV VMIs show a slightly wider tumor boundary than conventional images, and the tumor size and extent of tumor contact with the artery appear larger than in conventional images. Based on this hypothesis, the tumor boundary on low-keV VMIs may be more accurate than that on polychromatic images; however, further studies with pathologic correlation are needed.

Owing to the small number of patients in our study and limited life expectancy of PDAC, it is hard to say VMI_55_ of PVP is recommended than pancreatic-phase to reduce radiation dose in all PDAC patients. However, considering non-specific symptoms and very low incidence of PDAC, scanning pancreatic-phase in all patients suspicious for pancreatic disease should be reconsidered. In our study, only 65 patients were diagnosed with PDAC among 2,848 cases of pancreatic CT scan. Therefore, we propose using VMI_55_ of PVP for routine evaluation of the pancreas not to miss PDAC instead of an additional pancreatic-phase scan. If PDAC is detected in VMI_55_ of PVP and further image evaluation is needed for treatment, an additional pancreatic-phase CT scan or pancreas MRI could be helpful.

This study had some limitations. First, we identified study participants from CT reports, and patients whose PDAC was not visualized on CT images were not included. However, as the tumor was not visualized in the pancreatic-phase, the assessment of the noninferiority of VMIs of the PVP would not be affected. Second, we included a few patients who underwent surgery. Furthermore, because PDAC with arterial invasion is considered unresectable, only one patient showed vascular invasion. Thus, the performance of VMI for evaluating tumor size and arterial invasion could not be compared with pathologic data. Third, this was a single-center, retrospective study. As the kernel size affects image noise, SNR and CNR will differ across different institutions using different kernel sizes. Therefore, further multicenter studies with more operated patients with PDAC are needed to confirm the feasibility of replacing the pancreatic-phase scan with VMIs of PVP.

## Conclusion

In conclusion, for evaluating PDAC, VMIs at 40 keV and 55 keV of the PVP in dual-layer spectral CT demonstrated better objective tumor conspicuity and peripancreatic vascular enhancement than the pancreatic-phase image, and VMI at 55 keV showed better subjective tumor conspicuity than VMI at 40 keV. We recommend VMI at 55 keV of PVP as an alternative to the pancreatic-phase scan in patients suspicious of PDAC to reduce radiation exposure.

## Additional Files

The additional files for this article can be found as follows:

10.5334/jbsr.2798.s1 Supplementary file 1: Appendix. Materials and methods.

10.5334/jbsr.2798.s2 Supplementary file 2: Appendix. P values.
