# Supplementary material for: Dual-Layer Spectral CT of Pancreas Ductal Adenocarcinoma: Can Virtual Monoenergetic Images of the Portal Venous Phase Be an Alternative to the Pancreatic-Phase Scan?
Source: J Belg Soc Radiol. 2022 Sep 22;106(1):83. doi: 10.5334/jbsr.2798 (PMC9504095; doi:10.5334/jbsr.2798)
Supplement: Supplementary file 1: Appendix. — Materials and methods. [file jbsr-106-1-2798-s1.pdf]

Material/methods

Study Participants

Owing to the fact that it is a retrospective chart review study, our institutional review board approved this study (registry no. 2018AN0448), and waived the requirement for its informed consent.

The flow diagram of study participants were demonstrated in the Fig. 1. The inclusion criteria were PDAC patients who underwent pancreatic CT scan with dual-layer spectral CT. For patient inclusion, the CT reports of 2,848 consecutive patients who underwent pancreatic CT using dual-layer spectral CT between February 24, 2018 and January 29, 2020 were reviewed. Among CT reports of 2,848 patients, primary pancreatic cancer is mentioned in 77 patients. Sixty-five patients were included who were subsequently diagnosed with PDAC. The PDAC were pathologically diagnosed after pancreatic resection (n=8), EUS guided pancreatic biopsy or FNA (n=40), biopsy from hepatic or lung metastasis (n=7), or clinically diagnosed based on radiologic findings (MRI, follow-up CT), PET-CT, and elevated cancer antigen 19-9 (n=10). Twelve patients were not included because of the following reasons; final diagnosis of other tumors (n=4) or focal pancreatitis (n=2), normal pancreas on MRI and PET-CT (n = 1), follow-up loss (n = 5). The exclusion criteria were poor tumor conspicuity on CT scan for image anlysis, and one patient was excluded because of the tumor was not visualized on CT image owing to pancreatitis. This patient was diagnosed with PDAC after a pancreatectomy.

CT Protocol and Image Reconstruction

All CT scans were acquired using a dual-layer spectral CT (IQon Spectral CT; Philips Healthcare, Cleveland, OH). Pancreatic CT comprised a non-enhanced phase, a pancreatic-phase, and a PVP. The tube voltage was 120 kVp, and automated tube current modulation

(three-dimensional dose modulation) was applied. Intravenous contrast medium (Iomeron 350; Bracco, Milan, Italy) was injected at a rate of 3 mL/s for a total amount of 2 mL/kg body weight using a power injector system. The bolus tracking method was applied, and the pancreatic-phase scan started 27 s after the abdominal aorta reached 150 HU. The portal venous scan started 50 s after the abdominal aorta reached 150 HU. The upper abdomen was scanned in the non-enhanced and pancreatic-phases, and the whole abdomen and pelvis were scanned in the PVP. After the CT evaluation, the conventional polychromatic images of the pancreatic-phase were reconstructed at 120 kVp, and VMIs of the PVP were reconstructed at 40 keV (VMI_{40}), 55 keV (VMI_{55}), and 70 keV (VMI_{70}). VMI_{40} is the lowest energy setting provided by the software; VMI_{55} is equivalent to an 80-kVp (low kVp) image, and VMI_{70} is equivalent to a 120-kVp (standard kVp) image. A VMI reconstructed at >70 keV is not expected to show a better image contrast than a 120-kVp polychromatic image. An iterative reconstruction algorithm (iDose level 4) was used for the conventional polychromatic image of the pancreatic-phase and VMIs of the PVP. All conventional and VMI images were reconstructed axially with 3-mm slice thickness and 3-mm reconstruction intervals.

Image Analysis

For objective analysis, one radiologist with 5 years of experience in interpreting abdominal images performed image analysis using a workstation based on a picture archiving and communication system (INFINITT PACS version 3.0; Infinitt Healthcare Co., Seoul, South Korea). The investigator was aware of the patients' PDAC diagnoses. Circular ROIs were drawn in the normal pancreatic parenchyma, tumor, celiac trunk, portal vein, SMA, and SMV in the pancreatic-phase image and in VMI_{40}, VMI_{55}, and VMI_{70} of the PVP, respectively. The copy-and-paste function of the workstation was used to minimize the differences in the size and location of each ROI among the images. Efforts were made to place the ROI in the center

of the vascular lumen, and the vessel wall was not included. On each image in which the ROI was drawn in the vascular lumen, the other circular ROI was drawn in the most homogeneous portion of the paravertebral muscle. The average attenuation (AV) and standard deviation (SD) of the Hounsfield unit (HU) were recorded for each ROI.

The conspicuity of PDAC was evaluated using the tumor-to-pancreas CNR and attenuation difference.

$$\mathrm{CNR} = \frac{AV_P - AV_T}{(SD_P + SD_T)/2}$$

$$\text{Attenuation difference} = AV_P - AV_T$$

AV_P and SD_P are variables of the normal pancreatic parenchyma and AV_T and SD_T are tumor variables.

Peripancreatic vasculature enhancement was evaluated using SNR and CNR.

$$\mathrm{SNR} = \frac{AV_{VS}}{SD_{VS}}$$

$$\mathrm{CNR} = \frac{AV_{VS} - AV_{PM}}{SD_{PM}}$$

AV_{VS} and SD_{VS} are vessel variables and AV_{PM} and SD_{PM} are variables of the paravertebral muscle.

For subjective analysis, two radiologists (5 and 20 years of experience) independently analyzed the images. The investigators were aware of the patients' diagnoses but blinded to the original CT report, and analyzed each type of reconstructed image. The time interval between analysis of each type (the pancreatic-phase images, VMI_{40}, VMI_{55}, and VMI_{70}) of image was at least 2 weeks. The conspicuity of the PDAC was rated using a five-point scale (1=undiagnostic, 2=suboptimal, 3=average, 4=good, or 5=excellent). Tumor

conspicuity was defined as clear discernibility of the margin of the PDAC, in contrast to the normal pancreatic parenchyma. The heterogeneity of PDAC was rated on a three-point scale (0=homogeneous, 1=heterogeneous, or 2=cystic or necrotic). The largest PDAC size was measured on the axial images. Peripancreatic arterial invasion was evaluated using a three-point scale (0, no tumor contact; 1, tumor contact ≤180°; or 2, tumor contact >180°) for the celiac trunk, SMA, and splenic artery.

Statistical Analyses

The Wilcoxon signed-rank test and paired t-test were used to compare objective parameters and subjective tumor conspicuity. Cohen's simple kappa test was used to evaluate the inter-reader agreement of subjective parameters. The value of kappa for inter-reader agreement were interpreted as follows; 0–0.20 none, 0.21–0.39 minimal, 0.40–0.59 weak, 0.60–0.79 moderate, 0.80–0.90 strong, 0.91–1.00 almost perfect [1]. Bonferroni correction was performed for multiple comparisons, and a p-value of <.0083 was considered to indicate statistical significance when comparing objective parameters and subjective tumor conspicuity. In comparing tumor size, p<.017 was considered significant. All statistical analyses were performed using the SPSS software (version 25; SPSS).

References

1. McHugh ML. Interrater reliability: the kappa statistic. Biochem Med (Zagreb). 2012;22:276–282. doi:10.11613/BM.2012.031
