# Supplementary material for: Dual-Layer Spectral CT of Pancreas Ductal Adenocarcinoma: Can Virtual Monoenergetic Images of the Portal Venous Phase Be an Alternative to the Pancreatic-Phase Scan?
Source: J Belg Soc Radiol. 2022 Sep 22;106(1):83. doi: 10.5334/jbsr.2798 (PMC9504095; doi:10.5334/jbsr.2798)
Supplement: Supplementary file 2: Appendix. — P values. [file jbsr-106-1-2798-s2.pdf]

Supplementary table 1. p-Values of pairwise comparisons of parameters between the pancreatic-phase image and VMIs of the portal venous phase in evaluating pancreatic ductal adenocarcinoma and the peripancreatic vasculature with dual-layer spectral CT

	**Pancreatic-phase**			**VMI_{40}**		**VMI_{55}**
**Parameters**	**VMI_{40}**	**VMI_{55}**	**VMI_{70}**	**VMI_{55}**	**VMI_{70}**	**VMI_{70}**
Tumor-to-Pancreas Attenuation difference	<.001*	<.001*	.70	<.001*	<.001*	<.001*
Tumor-to-Pancreas CNR	<.001*†	<.001*†	<.001*†	<.001*†	<.001*†	<.001*
SNR						
Celiac trunk	<.001*	<.001*	.004*	<.001*	<.001*	<.001*
SMA	<.001*	<.001*	.14	<.001*†	<.001*	<.001*†
Portal vein	<.001*	<.001*	<.001*	<.001*	<.001*	<.001*
SMV	<.001*†	<.001*†	<.001*	<.001*	<.001*†	<.001*
CNR						
Celiac trunk	<.001*†	<.001*†	.35†	<.001*	<.001*	<.001*†
SMA	<.001*†	<.001*†	.36	<.001*†	<.001*	<.001*†
Portal vein	<.001	<.001*	<.001*†	<.001*†	<.001*	<.001*†
SMV	<.001*	<.001*†	<.001*†	<.001*†	<.001*	<.001*†

Unless otherwise indicated, the p-value is from paired t-test.

† p-value obtained from the Wilcoxon signed-rank test

*Statistically significant difference (p<.0083).

CNR: contrast-to-noise ratio; SNR: signal-to-noise ratio; VMI_{40}, VMI_{55}, and VMI_{70}: virtual monoenergetic images at 40, 55, and 70 keV of the portal venous phase, respectively; SMA: superior mesenteric artery; SMV: superior mesenteric vein.

Supplementary table 2. p-Values of pairwise comparisons of subjective parameters between the pancreatic-phase image and VMIs of the portal venous phase in evaluating pancreatic ductal adenocarcinoma and peripancreatic arteries with dual-layer spectral CT

**Subjective Parameters**	**Reader 1**	**Reader 2**
Tumor size		
VMI_{40}	.004*	.002*
VMI_{55}	.009*	.49
VMI_{70}	.04	.08
Tumor heterogeneity		
VMI_{40}	<.001*	<.001*
VMI_{55}	.001*	<.001*
VMI_{70}	.02	<.001*
Celiac trunk invasion		
VMI_{40}	.32	.003*
VMI_{55}	>.99	.007*
VMI_{70}	.53	.03
SMA invasion		
VMI_{40}	.01*	.01*
VMI_{55}	.008*	.003*
VMI_{70}	.16	.13
Splenic artery invasion		
VMI_{40}	.002*	.002*
VMI_{55}	.001*	.001*
VMI_{70}	.02	.03

† p-values from the Wilcoxon signed-rank test compared to the pancreatic-phase image. Bonferroni correction was applied for multiple comparisons, and $p<.017$ was considered significant.

* Statistically significant difference.

VMI_{40}, VMI_{55}, and VMI_{70}: virtual monoenergetic images at 40, 55, and 70 keV of the portal venous phase, respectively; SMA: superior mesenteric artery.
